# Immunosuppression as a Hallmark of Critical COVID-19: Prospective Study

**DOI:** 10.3390/cells10061293

**Published:** 2021-05-23

**Authors:** Elżbieta Kalicińska, Donata Szymczak, Aleksander Zińczuk, Barbara Adamik, Jakub Smiechowicz, Tomasz Skalec, Danuta Nowicka-Suszko, Monika Biernat, Aleksandra Bogucka-Fedorczuk, Justyna Rybka, Adrian Martuszewski, Waldemar Gozdzik, Krzysztof Simon, Tomasz Wróbel

**Affiliations:** 1 Department and Clinic of Hematology, Blood Neoplasms, and Bone Marrow Transplantation, Wroclaw Medical University, 50-367 Wroclaw, Poland; donata.szymczak@umed.wroc.pl (D.S.); monika.biernat@umed.wroc.pl (M.B.); aleksandra.bogucka-fedorczuk@umed.wroc.pl (A.B.-F.); justyna.rybka@umed.wroc.pl (J.R.); tomasz.wrobel@umed.wroc.pl (T.W.); 2 Department of Infectious Diseases and Hepatology, Wroclaw Medical University, 51-149 Wroclaw, Poland; aleksander.zinczuk@student.umed.wroc.pl (A.Z.); krzysztof.simon@umed.wroc.pl (K.S.); 3 Department of Forensic Medicine, Wroclaw Medical University, 50-345 Wroclaw, Poland; 4 Department of Anesthesiology and Intensive Therapy, Wroclaw Medical University, 50-556 Wroclaw, Poland; barbara.adamik@umed.wroc.pl (B.A.); jakub.smiechowicz@umed.wroc.pl (J.S.); tomasz.skalec@umed.wroc.pl (T.S.); waldemar.gozdzik@umed.wroc.pl (W.G.); 5 Department of Dermatology and Venereology and Allergology, Wroclaw Medical University, 50-368 Wroclaw, Poland; danuta.nowicka-suszko@umed.wroc.pl; 6 Students Scientific Association, Department and Clinic of Hematology, Blood Neoplasms, and Bone Marrow Transplantation, Wroclaw Medical University, 50-367 Wroclaw, Poland; adert123@interia.pl

**Keywords:** immunosuppression, lymphocyte subsets, T cells, regulatory T cells, NK cells, TCR γ/δ cells, TCRα/β, COVID-19, INFγ, TNFα, IL-2, IL-2/INFγ ratio

## Abstract

The dysregulation of both the innate and adaptive responses to SARS-CoV-2 have an impact on the course of COVID-19, and play a role in the clinical outcome of the disease. Here, we performed a comprehensive analysis of peripheral blood lymphocyte subpopulations in 82 patients with COVID-19, including 31 patients with a critical course of the disease. In COVID-19 patients who required hospitalization we analyzed T cell subsets, including Treg cells, as well as TCRα/β and γ/δ, NK cells, and B cells, during the first two weeks after admission to hospital due to the SARS-CoV-2 infection, with marked reductions in leukocytes subpopulations, especially in critically ill COVID-19 patients. We showed decreased levels of Th, Ts cells, Treg cells (both naïve and induced), TCRα/β and γ/δ cells, as well as CD16+CD56+NK cells in ICU compared to non-ICU COVID-19 patients. We observed impaired function of T and NK cells in critically ill COVID-19 patients with extremely low levels of secreted cytokines. We found that the IL-2/INFγ ratio was the strongest indicator of a critical course of COVID-19, and was associated with fatal outcomes. Our findings showed markedly impaired innate and adaptive responses in critically ill COVID-19 patients, and suggest that the immunosuppressive state in the case of a critical course of SARS-CoV-2 infection might reflect subsequent clinical deterioration and predict a fatal outcome.

## 1. Introduction

The SARS-CoV-2 pandemic affects all branches of medicine, and personal protective equipment helps to prevent the spread of the virus [1]. The significance of the SARS-CoV-2 infection results from the very wide spectrum of organ damage in the course of COVID-19, including not only respiratory and circulatory failure, but also neurological complications [2]. Novel SARS-CoV-2 tests and the availability of vaccines make the fight against COVID-19 more effective [3]. Insights into pathophysiology of the SARS-CoV-2 infection might facilitate the use of adjuvant supplements, such as agents improving the function of the immune system against the SARS-CoV-2 infection [4].

There is growing interest in the nature of the immune landscape in the course of the SARS-CoV-2 infection. The clinical significance of an imbalance in leukocyte homeostasis during COVID-19 is emphasized. Adequate immune responses in the SARS-CoV-2 infection depend not only on humoral, but also on the cellular system. It has been suggested that SARS-CoV-2 exposure provides virus-specific T cells, therefore the cellular response could serve as a more sensitive marker of viral response than antibodies [5]. Previous studies showed that a strong T cell response might develop sufficient immunity against COVID-19, even in the absence of antibodies [6]. It is well known that leukopenia is a hallmark of the SARS-Co-V-2 infection, especially in the case of a severe course of COVID-19 [7,8,9,10,11,12]. Decreased levels of lymphocytes were found to be a significant predictor of severe illness and mortality in COVID-19 [7,8,9,10,11,12]. However, the available data concerning the nature of a critical course of COVID-19 are contradictory. It is not established whether the poor outcome in the SARS-CoV-2 infection results from cytokine storm and excessive inflammatory host response, or rather from impairment of both innate and adaptive immunity.

In this prospective study, we analyzed the lymphocyte subsets in 82 Polish patients hospitalized due to moderate/severe and critical COVID-19. We compared the immune responses in non-ICU versus ICU patients with SARS-CoV-2 infection during the first two weeks after admission to hospital. We suggest that immune disturbances during the early stage of COVID-19 are crucial for the disease course and outcomes.

## 2. Materials and Methods

### 2.1. Study Population

We prospectively examined 82 patients with COVID-19, including 51 patients presenting with moderate/severe COVID-19 symptoms and 31 patients with critical COVID-19 hospitalized in the ICU. Criteria defining the clinical status of studied groups of patients were based on COVID-19 treatment guidelines [13]. Non-ICU COVID-19 patients include those with moderate or severe illness. Moderate illness was defined as evidence of lower respiratory disease during clinical assessment or imaging and oxygen saturation of (SpO2) >94% in room air [13]. Severe illness was defined as saturations below 94% in room air, a ratio of arterial partial pressure of oxygen to fraction of inspired oxygen (PaO2/FiO2) <300 mmHg, respiratory frequency >30 breaths/min, or lung infiltrates >50% [13]. All ICU COVID-19 patients had critical disease defined as respiratory failure, septic shock, and/or multiple organ dysfunction [13].

### 2.2. Study Design

A confirmed COVID-19 case was defined by a positive real-time reverse-transcriptase polymerase chain reaction (RT-PCR) assay using nasal and pharyngeal swab specimens. Demographic data, symptoms, signs, and laboratory measures were collected on admission to the hospital. Collected data did not include any personally identifiable information. The lymphocyte subsets in peripheral blood mononuclear cells (PBMCs) were assessed by FACS Canto II in 82 patients, including 31 patients requiring intensive care at several time points. The first was done after molecular confirmation of the SARS-CoV-2 infection (day 3 ± 2 days) and thorough the duration of the infection (day 7 ± 2 days, and day 12 ± 2 days). The following mouse anti-human monoclonal antibodies, all purchased from Becton Dickinson and Company (BD), San Jose, CA, were used for analysis: CD8 FITC, CD16 PE, CD4 PerCPCy5.5, CD56 PC-7, CD7 APC, CD14 APC-H7, HLA-DR V450, CD3 V500, TCRαβ FITC, CD5 PerCP-Cy5.5, TCRγδ PC-7, CD19 APC, CD8 APC-H7, CD4 V450, CD20 V450, CD38 PE, CD25 PE, CD127 PerCP-Cy5.5, CD45RO PC-7, CD45RA APC, CD3 APC-H7, CD45 V500. For FACS analysis, 20 mL of blood was collected in EDTA tubes (BD). Human PBMCs were separated using Ficoll-Hypaque (Sigma, St Louis, MO, USA). Cells were surface stained with the following fluorescence conjugated mouse anti-human monoclonal antibodies in one tube: CD8, CD16, CD4, CD56, CD7, CD14, HLA-DR, and CD3; in a second tube, cells were stained with the following antibodies: TCRαβ, CD38, CD5, TCRγδ, CD19, CD8, CD20, CD4, and CD3; and in the third tube, the following antibodies: CD8, CD25, CD127, CD45RO, CD45RA, CD3, CD4, and CD45. The evaluation of nucleated cells was carried out on an 8-color FACS Canto II flow cytometer (BD). The data were analyzed using BD FACSDiva software v 8.0—the gating strategy is shown in Figure S1.

The cytokines were measured using commercially available ELISA kits for TNF-α, INF-γ, IL-2, and IL-6 (R&D Systems, Biotechne, Minneapolis, MN, USA), according to the manufacturer’s instructions.

Written informed consents were obtained in non-ICU COVID-19 patients, whereas in ICU COVID-19 patients, consents were waived due to the state of unconsciousness in these patients, which was accepted by the Wroclaw Medical University ethics committee. The study was performed in accordance with the Wroclaw Medical University ethics committee (consent no. 315/2020 and 675/2020).

### 2.3. Statistical Analyses

Statistical analyses were carried out using Statistica 13.1 software for Windows. Categorical variables were presented as frequencies with percentages, whereas median and interquartile range (IQR) or range (min–max) were used to describe continuous variables. Categorical variables were compared using the χ2 test or Fisher’s exact test. Evaluation of data normality was performed using the Shapiro–Wilk test. Non-normally distributed continuous variables were compared using the Mann–Whitney test and Friedman test. For multiple comparisons, Wilcoxon signed-rank test with Bonferroni correction was used. Receiver operating characteristics (ROC) curves were used to assess the sensitivity, specificity, and area under the ROC curve (AUC) of the investigated parameters. *p* < 0.05 was considered statistically significant.

## 3. Results

### 3.1. Clinical Characteristics of Patients with COVID-19

We prospectively analyzed 82 patients with the SARS-CoV-2 infection, hospitalized in Polish medical centers between May 5 and December 2 2020, including 31 patients with a critical course of COVID-19 hospitalized in ICU. The baseline clinical characteristics of the studied patients included in the analysis are shown in Table 1.

The males comprised 65% of the ICU patients with COVID-19 in the study cohort and 45% of the patients with moderate/severe COVID-19. The most common comorbidity in both the studied groups of patients was arterial hypertension (51% and 68% in moderate/severe and critical COVID-19 patients, respectively) and diabetes mellitus type 2 (16% and 35% in moderate/severe and critical COVID-19 patients, respectively). Diabetes mellitus type 2 was the only comorbidity with significantly higher prevalence in critical COVID-19 patients (*p* = 0.039). Approximately one-third of the patients in both groups received ACE inhibitors/ARB antagonists and beta-blockers.

Among the non-ICU COVID-19 patients, 65% had a moderate illness and 35% had severe illness. All the ICU COVID-19 patients had acute respiratory distress syndrome (ARDS) and were classified as critically ill. Most of the non-ICU COVID-19 patients had a cough (59%) and fever (65%). Dyspnea was observed in 39% of the non-ICU COVID-19 patients. Among the ICU COVID-19 patients, 52% had signs of shock on admission, and 16% required extracorporeal membrane oxygenation (ECMO) application. The mortality rates were significantly higher in the ICU COVID-19 patients compared to the non-ICU COVID-19 patients (77% and 8%, respectively).

### 3.2. Clinical Features and Procedures in Patients with COVID-19

The laboratory data and applied therapies for the patients included in the study are given in Table 1. Lymphopenia was present in 67% of the non-ICU COVID-19 patients and 90% of the ICU COVID-19 patients. Significantly more pronounced lymphopenia was present in the ICU COVID-19 patients compared to the non-ICU COVID-19 patients (*p* < 0.001). Regarding other populations of leukocytes, the number of monocytes were significantly higher in the ICU COVID-19 patients compared to the non-ICU COVID-19 patients (*p* = 0.001). Anemia was observed in 31% of the non-ICU COVID-19 patients, and in 84% of the ICU COVID-19 patients. Significantly lower hemoglobin levels were observed in the ICU COVID-19 patients (*p* < 0.001). CRP was significantly higher in the ICU COVID-19 patients (*p* < 0.001).

Almost half of the ICU COVID-19 patients were treated with convalescent plasma (48%) and almost all of them were treated with steroids (dexamethasone) (94%). Approximately one-third of the ICU COVID-19 patients received remdesivir (viral RNA polymerase inhibitor) (29%). On the other hand, among the non-ICU COVID-19 patients, one-third were treated with convalescent plasma, and 22% of the non-ICU patients received remdesivir.

### 3.3. Serum Cytokine Profiles in COVID-19 Patients

The T lymphocytes and natural killer (NK) cells profiles were described, assessing the levels of IL-2, TNFα, and INFγ in the peripheral blood in both of the studied groups of patients with COVID-19. The data are shown in Table 2. The IL-2 levels in both the non-ICU and ICU COVID-19 patients were low (median level 0.29 pg/mL), almost within the normal ranges, whereas the levels of TNFα were slightly elevated in both the studied groups. The levels of INFγ were increased in both of the groups of patients, with significantly higher levels in the non-ICU compared to the ICU COVID-19 patients (*p* = 0.03). The IL-2/INFγ ratio was significantly higher in the critically ill COVID-19 patients (*p* < 0.001 and *p* = 0.007, respectively).

#### Serum Cytokine Profiles and Outcome in Patients with COVID-19

We have analyzed the profiles of the cytokines that are produced by Th1/Ts lymphocytes and NK cells in both of the studied COVID-19 groups. The data regarding secreted cytokines are shown in Table 3 No significant differences between the COVID-19 patients who recovered and died were observed in terms of TNFα and INFγ. The levels of IL-2 were higher in the COVID-19 patients who died, but due to very low values (within normal ranges), probably with no clinical significance. On the other hand, we have demonstrated that the IL-2/INFγ ratio, as a more reliable marker of T cell activity, was significantly higher in the COVID-19 patients with fatal outcomes (*p* < 0.001).

### 3.4. Lymphocyte Subpopulations Analysis in Patients with COVID-19

#### 3.4.1. Lymphocyte Subsets in ICU Versus Non-ICU COVID-19 Patients

The analysis of lymphocyte subsets in the patients with COVID-19 is shown in Table 4 and Figure S2. The cytometric comparison of relevant lymphocyte subsets is shown in Figure S2. The total number of T cells and NK cells were significantly decreased in the ICU patients with COVID-19 (340 cells/µL and 50 cells/µL, both *p* < 0.001).

Analyzing the different lymphocyte subpopulations, we have shown lower than normal levels of helper T (Th) cells (CD3+CD4+) in both of the studied groups of patients, with significantly reduced Th lymphocyte levels in the ICU COVID-19 patients (*p* = 0.002). The levels of Ts cells were significantly reduced only in the critical COVID-19 patients (*p* < 0.001).

The levels of naïve Th and naïve Ts cells were within the lower reference ranges in both the studied groups, but were significantly lower in the ICU COVID-19 patients compared to the non-ICU COVID-19 patients (*p* = 0.044 and *p* < 0.001, respectively). The levels of activated T cells (CD3+HLA-DR+) were higher in the critical COVID-19 patients (*p* = 0.007), but remained within the normal ranges.

Regarding regulatory T (Treg) cell populations, significantly lower levels were observed in the case of critical COVID-19 (*p* = 0.001), both in terms of naïve (CD45RA+CD3+CD4+CD25+CD127low+) and induced (CD45RO+CD3+CD4+CD25+CD127low+) subsets (*p* < 0.001 and *p* = 0.007, respectively).

The levels of TCRα/β and TCRγ/δ were decreased both in the moderate/severe COVID-19 and critical COVID-19 groups; however, the levels of both the populations were significantly lower in the ICU COVID-19 patients (both *p* < 0.001).

Among other lymphocyte subpopulations, the CD20+ B cells remained within the normal ranges in both the studied groups; however, they were significantly lower in the patients with critical COVID-19 (*p* = 0.001). On the other hand, CD16+CD56+ NK cells were below the normal ranges in the ICU COVID-19 patients, and were significantly lower compared to the non-ICU COVID-19 patients (*p* < 0.001).

#### 3.4.2. Lymphocyte Subpopulations and Outcome in Patients with COVID-19

The analysis of lymphocyte subsets in recovered versus died COVID-19 patients is given in Table 5. In our study, we have demonstrated that lower levels of Th and Ts cells were observed in the COVID-19 patients who died (both *p* = 0.001). Similarly, the patients with fatal outcomes were characterized by lower levels of naïve Th and Ts cells (*p* = 0.016 and *p* = 0.002, respectively). Regarding Treg cells, in the patients with COVID-19 who died, both the naïve and induced Treg cells were significantly decreased compared to in the patients who survived (*p* < 0.001 and *p* = 0.003, respectively). We have also shown that the TCRα/β and TCRγ/δ levels were significantly decreased in the patients with COVID-19 who died (both *p* < 0.001). Moreover, we have revealed lower levels of CD20+ B lymphocytes in the patients with fatal outcomes (*p* < 0.001). Regarding NK cells, the patients who died were characterized by significantly decreased levels of CD16+CD56+ NK cells (*p* < 0.001).

### 3.5. Changes in the Counts of Lymphocyte Subpopulations during COVID-19

We have assessed the changes in the lymphocyte subpopulations in the COVID-19 patients three times during the course of the infection, at 5–7 day intervals. The data are shown in Figure S3 (A–M) and Table S1.

The Th cells (CD3+CD4+) (Figure S3A) and Ts cells (CD3+CD8+) (Figure S3B) were significantly lower in the ICU COVID-19 patients compared to the non-ICU COVID-19 patients from the admission to hospital to day 7 (*p* = 0.002 and *p* < 0.001, respectively, for Th cells, *p* < 0.001 and *p* = 0.003, respectively, for Ts cells). In parallel, significantly lower levels of naïve Th (CD3+CD4+CD45RA+) (Figure S3L) and naïve Ts (CD3+CD8+CD45RA+) cells (Figure S3M) were observed from admission to day 7 in the ICU COVID-19 patients (*p* = 0.043 and *p* < 0.001, respectively, for naïve Th, and *p* < 0.001 and *p* = 0.006, respectively, for naïve Ts cells). Similarly, the levels of Treg cells (CD3+CD4+CD25+CD127low+) (Figure S3E) were significantly lower from admission to day 7 in the ICU COVID-19 patients (*p* = 0.001 and *p* < 0.001, respectively). Regarding the Treg subsets, naïve Treg cells (CD45RA+CD3+CD4+CD25+CD127low+) (Figure S3F) were significantly lower from the day of admission, through day 7, to day 12 in the ICU COVID-19 patients (*p* < 0.001, *p* < 0.001, *p* = 0.022, respectively), while induced Treg cells (CD45RO+CD3+CD4+CD25+CD127low+) (Figure S3G) were lower from admission to day 7 in the ICU COVID-19 patients (*p* = 0.007 and *p* < 0.001, respectively).

On the other hand, activated T cells (CD3+HLA-DR+) (Figure S3C) were significantly higher on the day of admission and on day 12 in the ICU COVID-19 patients (*p* = 0.007 and *p* = 0.002, respectively). Activated Ts cells (CD3+CD8+HLA-DR+) (Figure S3D) were significantly higher on day 12 in the ICU COVID-19 patients (*p* = 0.022).

TCRα/β and TCRγ/δ cells were significantly lower at the beginning of hospitalization and on day 7 in the ICU COVID-19 patients (all *p* < 0.001), with much lower levels regarding TCRγ/δ cells (Figure S3H,I).

Among other lymphocyte subsets, CD20+ B cells (Figure S3J) were significantly lower upon admission and on day 7 in the ICU COVID-19 patients (*p* = 0.001 and *p* < 0.001, respectively). The levels of CD16+CD56+ NK cells (Figure S3K) were also lower upon admission and on day 7 in the ICU COVID-19 patients (both *p* < 0.001).

### 3.6. Predictors of Critical Course of COVID-19

**Figure 1:**
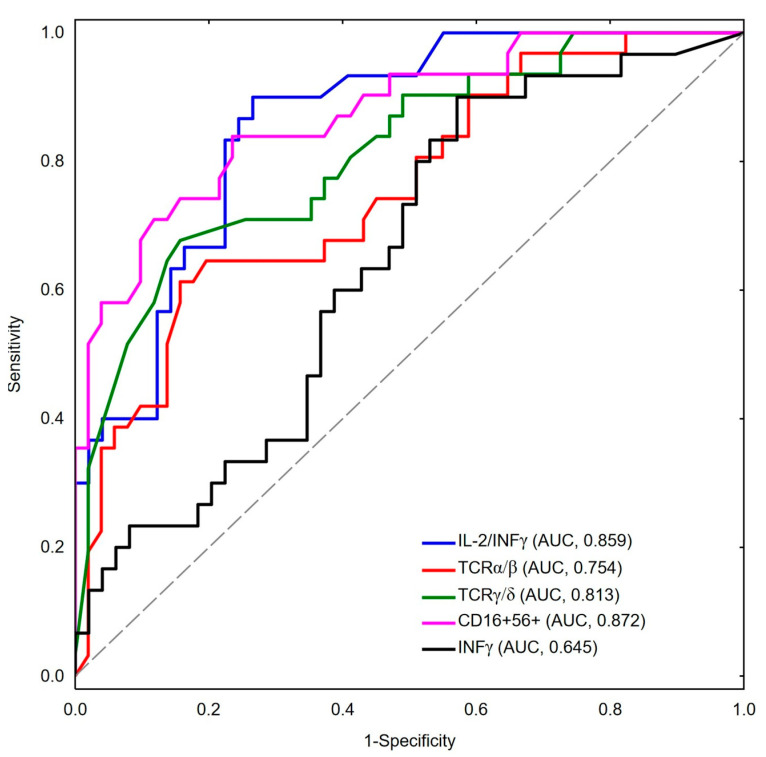
ROC curve analysis of different lymphocyte subsets and cytokines (IL-2/INFγ ratio—blue line, INFγ—black line, TCRα/β cells—red line, TCRγ/δ cells—green line, CD16+CD56+ NK cells—pink line).

ROC curve analysis was conducted to evaluate the relation of lymphocyte subsets and cytokines between moderate/severe versus a critical course of COVID-19. The IL-2/INFγ ratio was above the cut-off value of 0.09 for 27 ICU patients, which constitutes 90% of all the patients with a critical course of the infection. Based on the presented results, we singled out IL-2/INFγ ratio as the strongest marker for a critical course of COVID-19 among hospitalized patients with SARS-CoV-2 infection, with the area under the ROC curve (AUC) being 0.859 (95%CI:0.780–0.939), and with 73.5% specificity and 90% sensitivity. Moreover, our data have shown that TCRα/β, TCRγ/δ, CD16+56+NK cells, and INFγ levels appeared lower in the ICU patients with cut-off values of 0.375, 0.007, 0.080, and 5.97, and AUC values of 0.754 (95% CI: 0.646–0.862), 0.813 (95% CI: 0.717–0.909), 0.872 (95% CI: 0.794–0.951), and 0.645 (95% CI: 0.522–0.767). The presented results are shown in Figure 1.

## 4. Discussion

In our prospective study, we showed pronounced dysfunction of T cells and NK cells in COVID-19 patients with a critical course of the disease. Our research provides two major findings. First, patients with a critical course of COVID-19 are characterized by impaired innate and adaptive immunity. We found drastically lower levels of NK cells as well as a marked reduction in Th and Ts cells in the patients with COVID-19 who required intensive care, especially during the first week after molecular confirmation of the SARS-CoV-2 infection. We showed disturbed immunological homeostasis in critically ill COVID-19 patients who presented decreased levels of Treg cells. Our study established impaired T cell responses in the ICU COVID-19 patients with reduced expression of TCRα/β and extremely low expression of TCRγ/δ on the T cell surface. Secondly, we demonstrated profound dysfunction of T lymphocytes and NK cells in critically ill COVID-19 patients with diminished secretion of key cytokines such as IL-2, INFγ, and TNFα. Based on our results, a higher IL-2/INFγ ratio was a strong predictor of a critical course of COVID-19, and was significantly increased in COVID-19 patients who died.

The control of viral infections is based both on innate and adaptive immune responses [14]. NK cells, as a non-specific component of the immediate innate response, primarily control the acute phase of viral infection [14,15]. On the other hand, T cell responses are critical for long-term (>96 h) surveillance [14,15].

In our study, we showed a marked immunosuppressive state in critically ill COVID-19 patients. We found, especially in the ICU COVID-19 patients, numerically decreased subpopulations of T cells, which are crucial for host immunity, with parallel impaired function of T and NK cells in terms of the secretion of key cytokines. T cell loss and dysfunction might potentially lead to a more severe clinical course of the SARS-CoV-2 infection. Recent studies demonstrated decreased levels of Th and Ts lymphocytes [7,9,12,16,17,18,19,20] as well as NK cells, [7,8,9,16,17,18,19] with more profound reductions in the counts of Th cells in the severe course of COVID-19 [7,9].

Our analysis showed significantly lower levels of Th cells, as well as Ts cells and NK cells, in the critical COVID-19 patients. In the non-ICU COVID-19 patients, Ts cells (CD3+CD8+) and CD16+CD56+ NK cells remained within the normal ranges. In the ICU COVID-19 patients, both of the naïve subpopulations of Th and Ts cells were lower compared to the non-ICU COVID-19 patients, thus we can speculate that clonal expansion of T cells might be diminished with subsequent impairment of effective viral clearance and major tissue damage in the case of a critical course of the disease. Both T cells and NK cells provide anti-viral effects, not only by direct cytotoxic properties, but also by releasing anti-viral cytokines [14].

Interestingly in our study, not only the number, but also the function of Th, Ts, and NK cells were more effected in the critically ill COVID-19 patients. Protective, anti-viral patterns of T lymphocytes include the secretion of cytokines linked to multifunctionality, such as IL-2, TNFα, and INFγ [15]. These cytokines participate in T cell activation and have a role in maintaining particular T cell subsets [21]. Previous data suggest a failure to secrete sufficient amounts of effector cytokines by T cells in severe COVID-19 [22,23]. In terms of the secretion of cytokines (IL-2, TNFα, INFγ), which are produced mainly by Th1 cells, but also by Ts cells and NK cells, we observed very low levels of IL-2 (almost within the normal ranges), and only slightly elevated TNFα and INFγ levels in the ICU COVID-19 patients, whereas increased levels of INFγ were characteristic of the non-ICU COVID-19 patients. INFγ as a predominant cytokine in Th1 responses is a key anti-viral cytokine affecting both innate and adaptive immunity [24,25], and interfering directly with viral replication and indirectly with viral clearance [14,26]. In the course of the SARS-CoV-2 infection, it downregulates the expression of the ACE2 receptor [27], therefore possibly exerting protective effects of lung fibrosis in COVID-19 patients [28]. TNFα has also a significant impact on the proliferation and activation of naïve and effector T cells, and has a role in determining the pattern of the T cell pool affecting Treg cells [29]. Similarly, IL-2 is critical for the development, maintenance, and activity of Treg cells, [30] as well as it is known as T-cell growth factor, therefore reduction in its levels might result in disturbances of immune milieu.

Our findings indirectly demonstrated functional dysfunction of T cells and NK cells, which might indicate an exhaustion process, mostly concerning T cells, which was more pronounced in the critical COVID-19 patients. Exhausted T cells lose their capacity to secrete IL-2, INFγ, and TNFα [21,31,32]. Previous studies have shown that at the beginning of the exhaustion process T cells stop producing IL-2, next TNFα, and at the end INFγ [31,32]. In our study, in both of the COVID-19 groups of patients IL-2 was at the lowest level, TNFα was only slightly elevated, while INFγ was significantly higher in the non-ICU COVID-19 patients. These results suggest that the exhaustion process of lymphocytes concerns both the COVID-19 groups of patients; however, it is more profound in critical COVID-19 patients in whom all cytokines, including INFγ levels, are decreased. Therefore, we can speculate that the patients with critical COVID-19 have a weaker anti-viral immune response. Because IL-2 and INFγ might have an additional potential impact on B cell activation [33], and in consequence might have a role in the humoral immune response, we have assessed the clinical value of the IL-2/INFγ ratio—on the one hand, as an indicator of impaired both T and NK cells function, and on another, as an indirect marker of B cell response in the course of COVID-19. We have demonstrated that an increased IL-2/INFγ ratio was the strongest indicator of a critical course of COVID, and was associated with the mortality of the disease. Previous data similarly showed a higher proportion of INFγ−producing Th cells in patients with moderated COVID-19 than in patients with severe disease [12,23]. The dysfunction of T cells might impair the B cell compartment and, as we showed in our study, B cell levels were also lower in the critical COVID-19 patients.

In the critical COVID-19 patients, we demonstrated higher levels of activated T cells during the first two weeks after molecular confirmation of the SARS-CoV-2 infection. Excessive T cell activation might serve as a potential driver of COVID-19 [12,23,34,35,36]. Hyperactivation of T cells might result in functional impairment and possibly lead to the subsequent exhaustion in ICU COVID-19 patients [22,23]. Moreover, it might be associated with a reduction in circulatory Treg cells [9,12,37], which was described in our study and observed in previous reports [9,12,37].

The subsequent lymphocyte subsets involved in limiting the excessive host antiviral response are Treg cells. Treg cells play a key role in the control of the immune response through sustaining immunological homeostasis [38,39]. They have a crucial function in the regulation of the magnitude of the immune response to infection [8,40,41]. In the critical COVID-19 patients, we demonstrated significantly decreased levels of naïve and induced Treg cells. Some hypotheses concerning low Treg levels in COVID-19 patients suggested that low IL-2 parallel with high IL-6 in severe cases leads to enhanced apoptosis of Treg cells [42]. In our study, as mentioned above, we showed reduced levels of circulating IL-2 and increased IL-6 in the case of a critical course of COVID-19, which might result, according to the aforementioned hypothesis, in decreased Treg cells in ICU COVID-19 patients. The lower levels of naïve Treg cell subpopulations that were observed during the first two weeks after molecular confirmation of the SARS-CoV-2 infection in the ICU COVID-19 patients, might be associated with lower multifunctionality of T cells upon SARS-CoV-2 exposition. Decreased levels of Treg cells in the ICU COVID-19 patients might be associated with serious impairment in the immunoregulatory pathway of T cell-mediated immunity engendering immune cell activation [9]. Moreover, a diminished number of induced Treg cells in the ICU COVID-19 patients might lead to exacerbation of systemic inflammation and potentially might have a negative impact on the limitation of tissue immunopathology, which results in the lung injury observed in the ICU COVID-19 patients [43,44]. Observed low levels of Treg cells might be responsible for triggering the systemic inflammation present in the severe SARS-CoV-2 infection [43,44]. In our study, we confirmed higher levels of inflammatory markers—C-reactive protein and IL-6—in critically ill COVID-19 patients compared to non-ICU COVID-19 patients. Therefore, we can speculate that a suppressed T cell response might be the main driver of pathological inflammation in the critical course of the SARS-CoV-2 infection due to the dysregulated T cell–cytokine axis. Taking into account T cell responses, in the ICU COVID-19 patients we observed significantly decreased levels of TCRα/β and TCRγ/δ compared to the non-ICU COVID-19 patients. Immune responses to infections are mediated by increased expression of α/β and γ/δ cells on the T cell surface [45]. In our study, both levels of TCRα/β and TCRγ/δ were decreased in the ICU COVID-19 patients during the first week after molecular confirmation of the SARS-CoV-2 infection. Interestingly, the levels of TCRγ/δ were drastically lower in the patients with a critical course of COVID-19. Additionally, in the ICU COVID-19 patients, during the first week after confirmation of the infection, we observed lower levels of antigen CD25 on the T cell surface, which is a marker of γ/δ T cell activation. Therefore, we can speculate that not only the levels, but also the function was impaired in the critically ill COVID-19 patients. These results are comparable with our previous study, which showed decreased and dysfunctional TCRγ/δ in hematological patients with COVID-19 [46]. It is important to notice that both critically ill patients with COVID-19 as well as patients with hematological malignancies are characterized by their impaired ability to eliminate pathogens. The presented results clearly show that during the crucial, first week of a critical course of COVID-19, γ/δ T cells might become exhausted with impaired responsiveness to viral antigens.

The study limitations should be mentioned. We did not assess lymphocyte changes during the whole course of COVID-19, as well as we did not explore the potential impact of the applied treatments on lymphocyte subsets nor cytokines variations, because our observations concern only the early phase of the SARS-CoV-2 infection. This topic requires further investigation. However, as we showed in our study, that the early changes in lymphocytes are crucial in determining the disease course and outcome. Secondly, we did not assess the potential infiltration of key organs by leukocytes and inflammatory cells, so we cannot exclude the possible migration of immune cells into inflamed lungs in critical COVID-19 patients, as well as we did not have data concerning virus load in the ICU COVID-19 patients versus the non-ICU COVID-19 patients. Further investigations are needed to assess the potential impact of viral load on the lymphocyte milieu in COVID-19 patients.

## 5. Conclusions

Our study showed impairment of host protective immunity in critically ill COVID-19 patients, with respect to both innate and adaptive responses. Early disturbances of the lymphocytes landscape in ICU COVID-19 patients included numerically reduced levels of Th and Ts cells, Treg cells, and NK cells parallel with functional failure of these cells. T cell responses were ineffective in terms of markedly reduced TCRα/β and γ/δ, with a concomitant decrease in CD3+CD25+T cells in ICU COVID-19 patients. Early immunological failure of the host response in the case of a critical course of COVID-19 impedes the control of viral infection and leads to a fatal outcome.

## Figures and Tables

**Table 1:** Baseline clinical characteristics of patients with COVID-19.

Variable	All Patients, *n* = 82	Non-ICU COVID-19 Patients, *n* = 51	ICU COVID-19 Patients, *n* = 31	*p*
Age (years)	62 ± 16	62 ± 16	63 ± 15	0.848
Male, *n* (%)	43 (52%)	23 (45%)	20 (65%)	0.25
Comorbidities				
Hypertension, *n* (%)	47 (57%)	26 (51%)	21 (68%)	0.14
Diabetes mellitus, *n* (%)	19 (23%)	8 (16%)	11 (35%)	0.039
Heart failure, *n* (%)	11 (13%)	7 (14%)	4 (13%)	0.91
Coronary artery disease, *n* (%)	12 (15%)	8 (16%)	4 (13%)	0.73
Renal failure, *n* (%)	3 (4%)	3 (6%)	0 (0%)	0.29
Stroke/TIA, *n* (%)	5 (6%)	3 (6%)	2 (6%)	1
Concomitant medications				
ACE-I/ARB, *n* (%)	20 (24%)	11 (22%)	9 (29%)	0.18
Beta blockers, *n* (%)	26 (32%)	18 (35%)	8 (26%)	0.78
Calcium blockers, *n* (%)	15 (18%)	11 (22%)	4 (13%)	0.57
Diuretics, *n* (%)	18 (22%)	15 (29%)	3 (10%)	0.09
White blood cells (×10^3^/µL)]	8.2 (5.3–12.7)	6.0 (4.2–8.4)	13.8 (9.7–17.0)	<0.001
Lymphocytes (×10^3^/µL)	1.0 (0.6–1.5)	1.1 (0.9–1.7)	0.6 (0.4–0.9)	<0.001
Neutrophiles (×10^3^/µL)	5.6 (3.3–10.8)	3.7 (2.7–5.5)	11.6 (9.1–15.7)	<0.001
Basophiles (×10^3^/µL)	0.02 (0.01–0.1)	0.02 (0.01–0.03)	0.1 (0.04–0.2)	<0.001
Eosinophiles (×10^3^/µL)	0.03 (0–0.1)	0.02 (0–0.07)	0.1 (0–0.36)	0.045
Monocytes (×10^3^/µL)	0.6 (0.5–1.25)	0.6 (0.5–0.7)	1.9 (0.5–4.6)	0.001
Hemoglobin (g/dl)	12.9 (11.4–14.2)	13.7 (12.4–14.6)	11.2 (10.7–12.8)	<0.001
Platelets (×10^3^/µL)	228 (171–308)	220 (154–288)	238 (188–369)	0.11
Inflammatory markers				
CRP (mg/L)	46.0 (16.6–118)	28.6 (7.2–77.1)	107.5 (28–194)	<0.001
IL-6, pg/mL (normal ranges: 0.5–3.9)	63 (17–127)	26 (8–91)	84 (38–209)	0.001
COVID-19 severity	-			-
Moderate, *n* (%)		33 (65%)	0 (0%)	
Severe, *n* (%)		18 (35%)	0 (0%)	
Critical, *n* (%)		0 (0%)	31 (100%)	
Severity of disease in ICU patients (points)	-	-		-
APACHE II			15 (12–22)	
SOFA			9 (5–10)	
Time of SARS-CoV-2 infection	12 (9–19)	11 (9–16)	17 (9–20)	0.16
Clinical outcome, death, n (%)	28 (34%)	4 (8%)	24 (77%)	<0.001
Treatment				
Oxygen therapy, *n* (%)	20 (24%)	20 (39.2%)	0 (0%)	1
High-Flow Nasal Oxygen, *n* (%)	7 (9%)	7 (14%)	0 (0%)	1
Mechanical ventilation, *n* (%)	31 (38%)	0 (0%)	31 (100%)	1
Remdesivir, *n* (%)	20 (24%)	11 (22%)	9 (29%)	0.445
Convalescent plasma, *n* (%)	30 (37%)	15 (29%)	15 (48%)	0.096
Tocilizumab, *n* (%)	4 (5%)	3 (6%)	1 (3%)	1
Steroids, *n* (%)	32 (39%)	3 (6%)	29 (94%)	<0.001
Azytromycin, *n* (%)	36 (44%)	35 (69%)	1 (3%)	<0.001

**Table 2:** Comparison of cytokines pattern between non-ICU and ICU patients with COVID-19.

Cytokines Levels	Normal Ranges (pg/mL)	All Patients (*n* = 82)	Non-ICU COVID-19 Patients (*n* = 51)	ICU COVID-19 Patients (*n* = 31)	*p*
IL-2, pg/mL	0–0.4	0.3 (0.2–0.5)	0.2 (0.1–0.3)	0.4 (0.3–0.8)	0.001
TNFα, pg/mL	0.8–1.7	1.9 (1.3–2.9)	1.9 (1.4–2.7)	2.1 (1.2–3.4)	0.56
INFγ, pg/mL	0–1.0	2.7 (0.9–9)	4.5 (1–11)	1.9 (0.8–4.7)	0.032
IL-2/INFγ	n/a	0.09 (0.03–0.3)	0.03 (0.02–0.1)	0.25 (0.13–0.7)	<0.011

**Table 3:** Comparison of cytokines pattern between patients with COVID-19 who recovered and died.

Cytokines Levels	Normal Ranges (pg/mL)	All Patients (*n* = 82)	Recovered COVID-19 Patients (*n* = 54)	Died COVID-19 Patients (*n* = 28)	*p*
IL-2, pg/mL	0–0.4	0.3 (0.16–0.5)	0.2 (0.1–0.3)	0.4 (0.3–0.8)	0.001
TNFα, pg/mL	0.8–1.7	1.9 (1.3–2.9)	1.8 (1.3–2.6)	2.5 (1.5–3.6)	0.06
INFγ, pg/mL	0–1.0	2.7 (0.9–9)	3 (1–11)	1.9 (0.8–5.6)	0.41
IL-2/INFγ	n/a	0.09 (0.03–0.3)	0.04 (0.02–0.2)	0.24 (0.1–0.8)	<0.011
IL-6, pg/mL	0.5–3.9	63 (17–127)	26 (9–77)	118 (46–261)	<0.001

**Table 4:** Comparison of lymphocyte subsets between non-ICU and ICU patients with COVID-19.

Lymphocyte Subsets	Normal Ranges /µL	All Patients (*n* = 82)	Non-ICU COVID-19 Patients (*n* = 51)	ICU COVID-19 Patients (*n* = 31)	*p*
T cells (CD3+)/µL	617–2383	503 (340–798)	620 (450–1010)	340 (250–600)	<0.001
Helper T cells (CD3+CD4+)/µL	424–1513	340 (210–536)	374 (279–588)	245 (150–395)	0.002
Suppressor T cells (CD3+CD8+)/µL	101–955	152 (95–270)	191 (116–360)	100 (68–172)	<0.001
Naïve helper T cells (CD3+CD4+CD45RA+)/µL	84–761	160 (98–275)	163 (111–367)	125 (64–223)	0.044
Naïve suppressor T cells (CD3+CD8+CD45RA+)/µL	100–400	99 (68–195)	128 (86–238)	70 (50–110)	<0.001
Activated T cells (CD3+HLA-DR+)/µL	30–200	34 (21–60)	30 (18–43)	44 (28–100)	0.007
Activated suppressor T cells (CD3+CD8+HLA-DR+)/µL	30–180	17 (7–33)	13 (7–27)	22 (7–55)	0.18
Regulatory T cells (CD3+CD4+CD25+CD127low+)/µL	30–120	21 (16–36)	26 (18–44)	17 (12–25)	0.001
Naïve regulatory T cells (CD45RA+CD3+CD4+CD25+CD127low+)/µL	9–36	3 (1–9)	4 (2–10)	2 (1–4)	<0.001
Induced regulatory T cells (CD45RO+CD3+CD4+CD25+CD127low+)/µL	21–84	18 (12–27)	22 (14–33)	14 (10–20)	0.007
TCRα/β/µL	573–2216	488 (320–780)	597 (412–940)	330 (250–598)	<0.001
TCRγ/δ/µL	30–119	11 (5–44)	23 (8–60)	4 (2–13)	<0.001
CD25+CD3+/µL	7–94	64 (40–95)	75 (56–119)	40 (26–71)	0.001
B cells (CD19+)/µL	31–527	129 (68–198)	128 (67–220)	130 (70–185)	0.85
B cells (CD19+CD20+)/µL	66–528	265 (167–375)	300 (197–423)	190 (134–310)	0.001
NK cells (CD16+CD56+)/µL	110–678	88 (47–192)	145 (81–217)	40 (17–77)	<0.001

**Table 5:** Comparison of lymphocyte subsets between patients with COVID-19 who recovered and died.

Lymphocyte Subsets	Normal Ranges /µL	All Patients (*n* = 82)	Recovered COVID-19 Patients (*n* = 54)	Died COVID-19 Patients (*n* = 28)	*p*
T cells (CD3+)/µL	617–2383	503 (340–798)	600 (450–990)	335 (268–535)	<0.001
Helper T cells (CD3+CD4+)/µL	424–1513	340 (210–536)	390 (279–584)	225 (154–364)	0.001
Suppressor T cells (CD3+CD8+)/µL	101–955	152 (95–270)	186 (116–352)	103 (70–170)	0.001
Naïve helper T cells (CD3+CD4+CD45RA+)/µL	84–761	160 (98–275)	167 (118–280)	120 (63–204)	0.016
Naïve suppressor T cells (CD3+CD8+CD45RA+)/µL	100–400	99 (68–195)	126 (78–230)	79 (53–105)	0.002
Activated T cells (CD3+HLA-DR+)/µL	30–200	34 (21–60)	33 (20–54)	35 (23–71)	0.4
Activated suppressor T cells (CD3+CD8+HLA-DR+)/µL	30–180	17 (7–33)	17 (8–30)	13 (6–41)	0.81
Regulatory T cells(CD3+CD4+CD25+CD127low+)/µL	30–120	21 (16–36)	26 (19–43)	17 (12–21)	<0.001
Naïve regulatory T cells (CD45RA+CD3+CD4+CD25+CD127low+)/µL	9–36	3 (1–9)	4 (2–10)	2 (1–3)	<0.001
Induced regulatory T cells (CD45RO+CD3+CD4+CD25+CD127low+)/µL	21–84	18 (12–27)	21 (14–32)	14 (10–20)	0.003
TCRα/β/µL	573–2216	488 (320–780)	588 (412–940)	325 (255–532)	<0.001
TCRγ/δ/µL	30–119	11 (5–44)	20 (8–53)	5 (2–14)	<0.001
CD25+CD3+/µL	7–94	64 (40–95)	75 (60–116)	40 (27–57)	<0.001
B cells (CD19+)/µL	31–527	129 (68–198)	130 (70–235)	101 (67–155)	0.24
B cells (CD19+CD20+)/µL	66–528	265 (167–375)	300 (220–420)	165 (125–285)	<0.001
NK cells (CD16+CD56+)/µL	110–678	88 (47–192)	135 (78–204)	42 (18–66)	<0.001

## Data Availability

The data are available in corresponding author.

## Supplementary Materials

The following are available online at https://www.mdpi.com/article/10.3390/cells10061293/s1, Figure S1: the gating strategy, Figure S2: comparison lymphocyte subsets between ICU COVID-19 patients and non-ICU COVID-19 patients, Figure S3: changes in the counts of lymphocyte subpopulations during COVID-19, Table S1: changes in the counts of lymphocyte subpopulations during SARS-CoV-2 infection in non-ICU versus ICU COVID-19 patients.
