# Supplementary material for: Immunosuppression as a Hallmark of Critical COVID-19: Prospective Study
Source: Cells. 2021 May 23;10(6):1293. doi: 10.3390/cells10061293 (PMC8224622; doi:10.3390/cells10061293)
Supplement: Supplementary file 1 [file cells-10-01293-s001.zip › Supp fig 3.pdf]

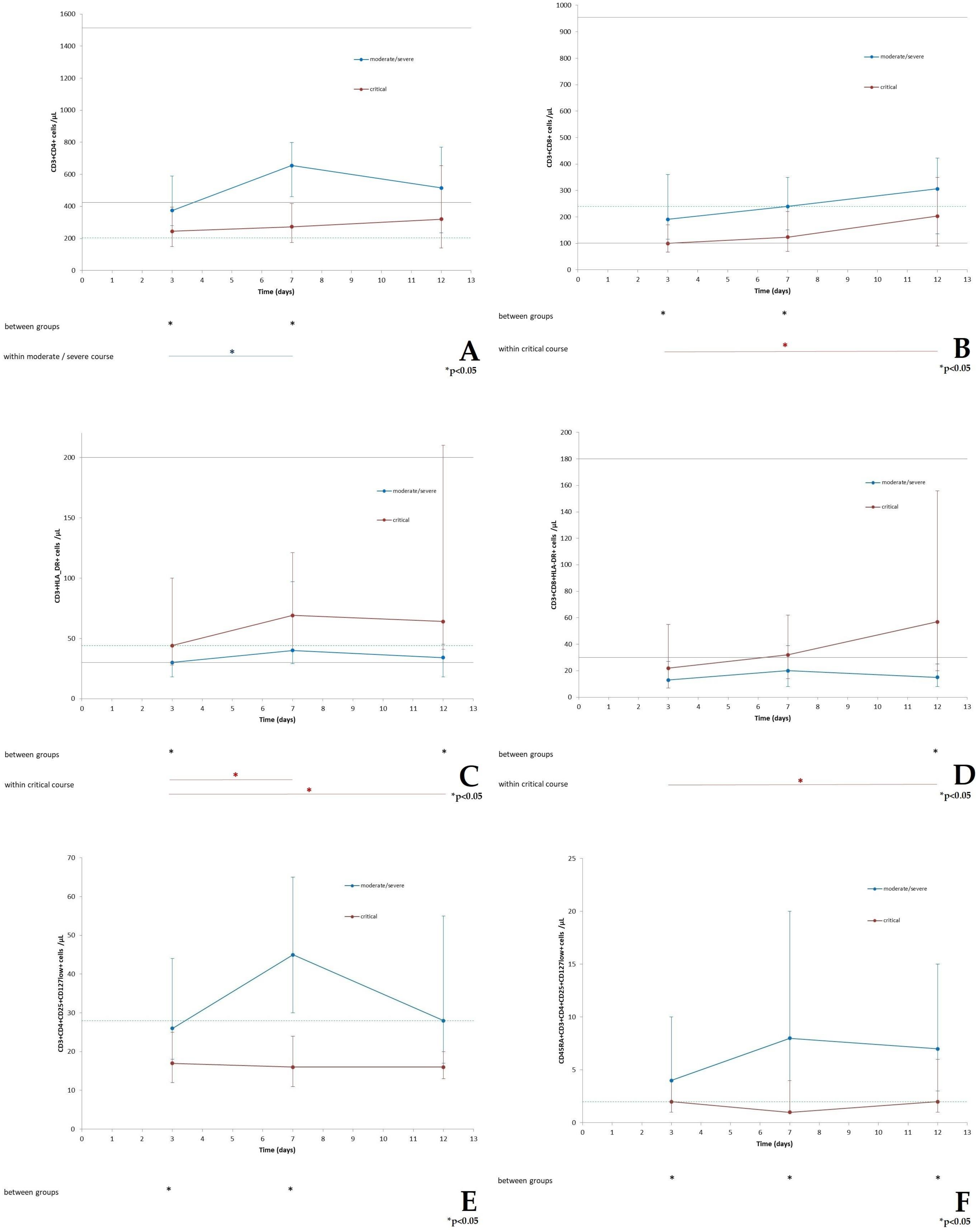

moderate/severe
critical
CD3+CD4+ cells /µL
Time (days)
between groups
within moderate / severe course
A
*p<0.05
CD3+CD8+ cells /µL
Time (days)
between groups
within critical course
B
*p<0.05
CD3+HLA_DR+ cells /µL
Time (days)
between groups
within critical course
C
*p<0.05
CD3+CD8+HLA-DR+ cells /µL
Time (days)
between groups
within critical course
D
*p<0.05
CD3+CD4+CD25+CD127low+ cells /µL
Time (days)
between groups
E
*p<0.05
CD45RA+CD3+CD4+CD25+CD127low+cells /µL
Time (days)
between groups
F
*p<0.05

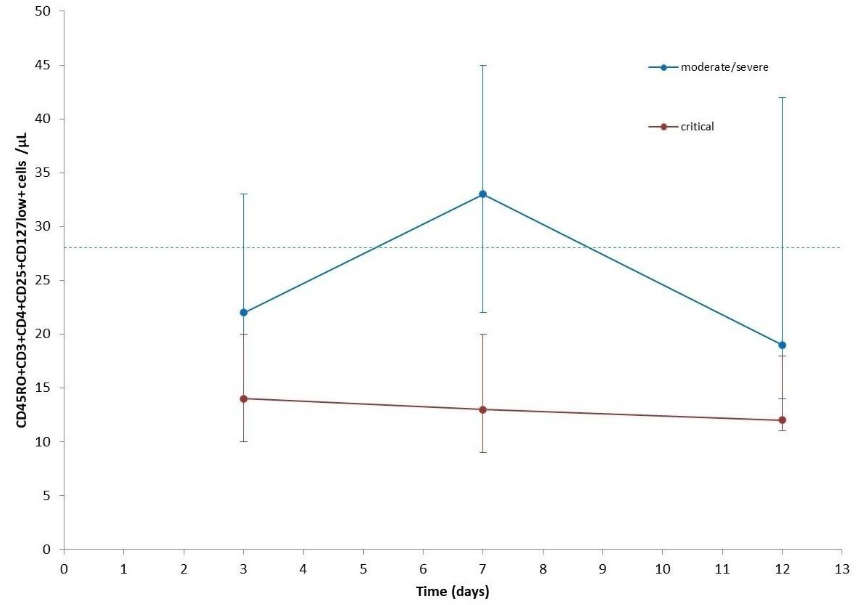

between groups * *

G

*p<0.05

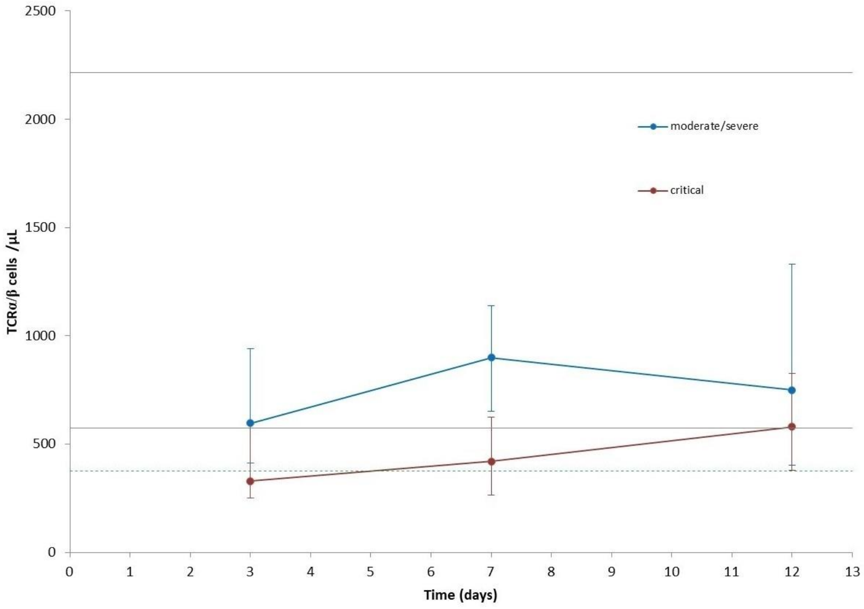

between groups * *

within critical course *

H

*p<0.05

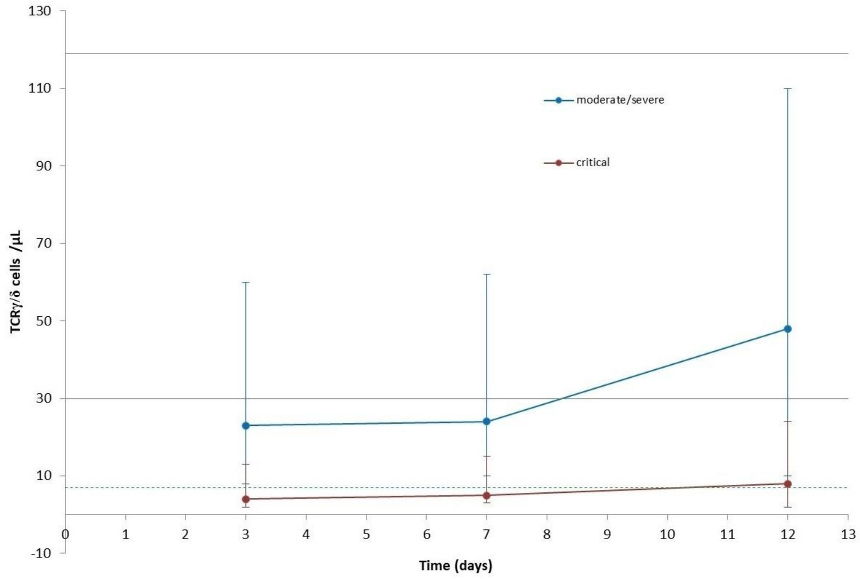

between groups * *

I

*p<0.05

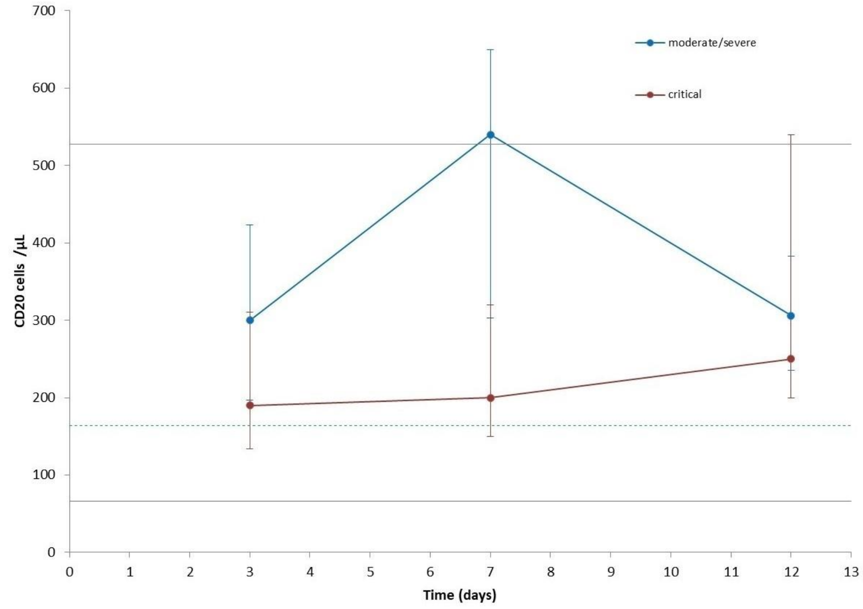

between groups * *

within critical course * *

J

*p<0.05

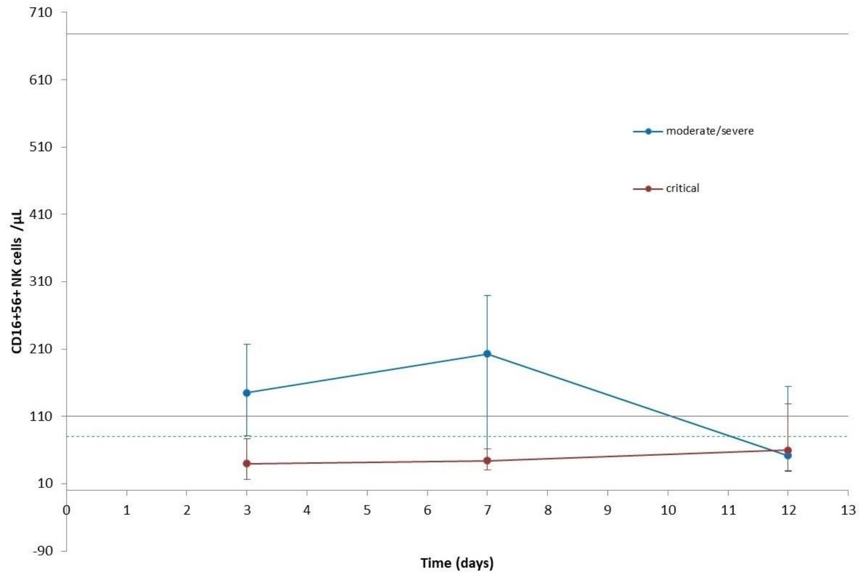

between groups * *

K

*p<0.05

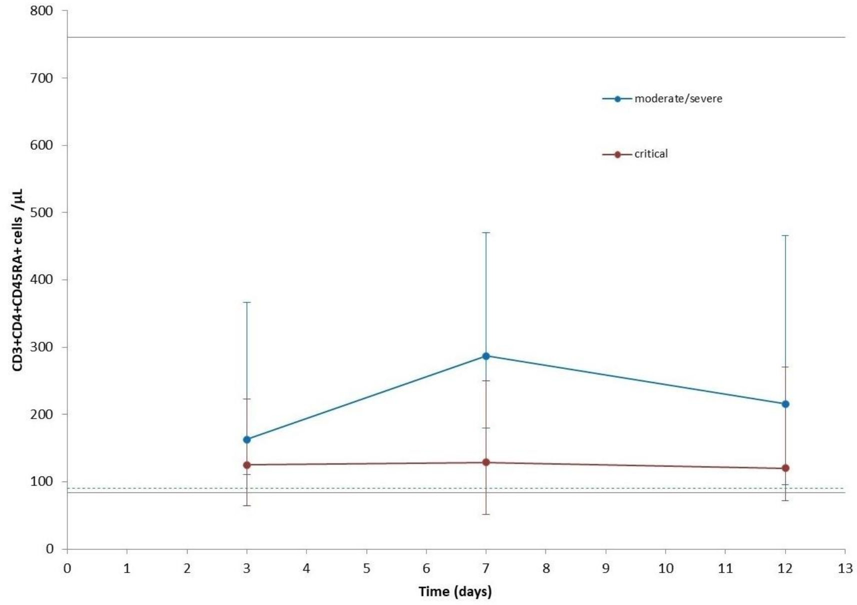

between groups * *

L

*p<0.05

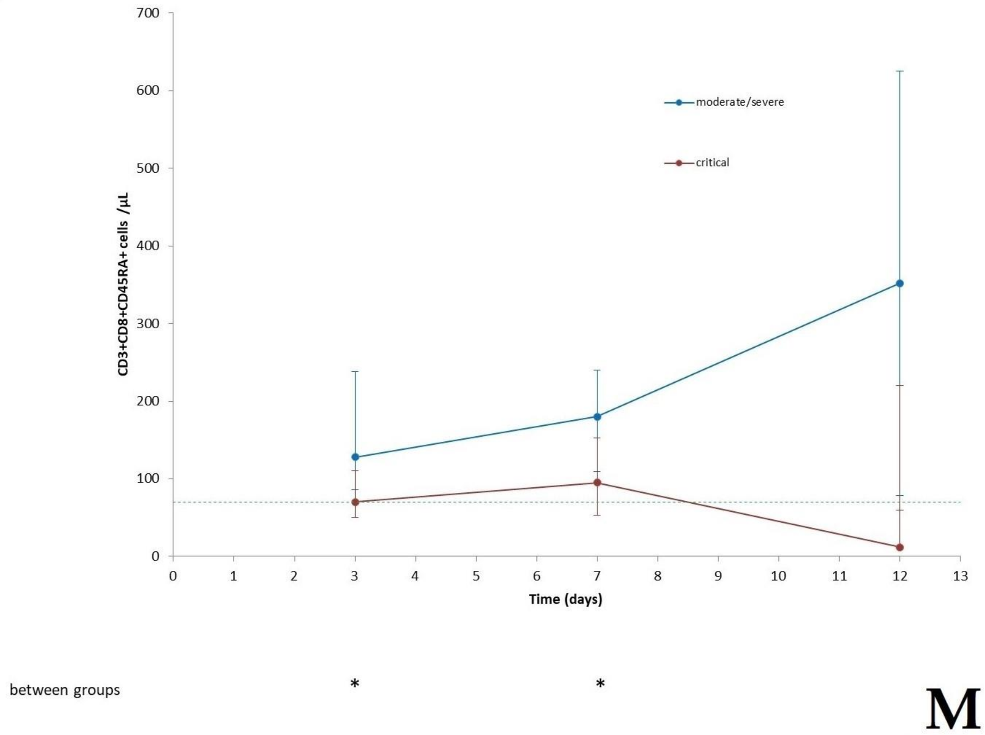

M

*p<0.05

Figure S3. Changes in the counts of lymphocyte subpopulations during COVID-19.

A – Changes in Th cells (CD3+CD4+) during the course of SARS-CoV-2 infection in moderate/severe (blue line) versus critical (red line) COVID-19 patients. Statistical significance between studied groups and during the COVID-19 course in particular groups of patients marked with *.

B – Changes in Ts cells (CD3+CD8+) during the course of SARS-CoV-2 infection in moderate/severe (blue line) versus critical (red line) COVID-19 patients. Statistical significance between studied groups and during the COVID-19 course in particular groups of patients marked with *.

C – Changes in activated T cells (CD3+HLA-DR+) during the course of SARS-CoV-2 infection in moderate/severe (blue line) versus critical (red line) COVID-19 patients. Statistical significance between studied groups and during the COVID-19 course in particular groups of patients marked with *.

D – Changes in activated Ts cells (CD3+CD8+HLA-DR+) during the course of SARS-CoV-2 infection in moderate/severe (blue line) versus critical (red line) COVID-19 patients. Statistical significance between studied groups and during the COVID-19 course in particular groups of patients marked with *.

E – Changes in Treg cells (CD3+CD4+CD25+CD127low+) during the course of SARS-CoV-2 infection in moderate/severe (blue line) versus critical (red line) COVID-19 patients. Statistical significance between studied groups marked with *.

F – Changes in naïve Treg cells (CD45RA+CD3+CD4+CD25+CD127low+) during the course of SARS-CoV-2 infection in moderate/severe (blue line) versus critical (red line) COVID-19 patients. Statistical significance between studied groups marked with *.

G – Changes in induced Treg cells (CD45RO+CD3+CD4+CD25+CD127low+) during the course of SARS-CoV-2 infection in moderate/severe (blue line) versus critical (red line) COVID-19 patients. Statistical significance between studied groups marked with *.

H – Changes in TCRα/β during the course of SARS-CoV-2 infection in moderate/severe (blue line) versus critical (red line) COVID-19 patients. Statistical significance between studied groups and during the COVID-19 course in particular groups of patients marked with *.

I – Changes in TCRγ/δ during the course of SARS-CoV-2 infection in moderate/severe (blue line) versus critical (red line) COVID-19 patients. Statistical significance between studied groups marked with *.

J – Changes in activated CD20+ B cells during the course of SARS-CoV-2 infection in moderate/severe (blue line) versus critical (red line) COVID-19 patients. Statistical significance between studied groups and during the COVID-19 course in particular groups of patients marked with *.

K – Changes in CD16+CD56+ NK cells during the course of SARS-CoV-2 infection in moderate/severe (blue line) versus critical (red line) COVID-19 patients. Statistical significance between studied groups marked with *.

L – Changes in naïve Th cells (CD3+CD4+CD45RA+) during the course of SARS-CoV-2 infection in moderate/severe (blue line) versus critical (red line) COVID-19 patients. Statistical significance between studied groups patients marked with *.

M – Changes in naïve Ts cells (CD3+CD8+CD45RA+) during the course of SARS-CoV-2 infection in moderate/severe (blue line) versus critical (red line) COVID-19 patients. Statistical significance between studied groups marked with *.
