# Supplementary material for: Immunosuppression as a Hallmark of Critical COVID-19: Prospective Study
Source: Cells. 2021 May 23;10(6):1293. doi: 10.3390/cells10061293 (PMC8224622; doi:10.3390/cells10061293)
Supplement: Supplementary file 1 [file cells-10-01293-s001.zip › Table S1.pdf]

Table S1. Changes in the counts of lymphocyte subpopulations during SARS-CoV-2 infection in non-ICU versus ICU COVID-19 patients.

Lymphocyte subsets	On admission	Day 7	Day 12
Helper T cells (CD3+CD4+)/μL in non-ICU	497	657	565
Helper T cells (CD3+CD4+)/μL in ICU	292	307	396
p	*	*	
Suppressor T cells (CD3+CD8+)/μL in non-ICU	250	283	319
Suppressor T cells (CD3+CD8+)/μL in ICU	119	167	245
p	*	*	
Naïve helper T cells (CD3+CD4+CD45RA+)/μL in non-ICU	261	338	321
Naïve helper T cells (CD3+CD4+CD45RA+)/μL in ICU	163	156	200
p	*	*	
Naïve suppressor T cells (CD3+CD8+CD45RA+)/μL in non-ICU	180	201	351
Naïve suppressor T cells (CD3+CD8+CD45RA+)/μL in ICU	86	120	153
p	*	*	
Activated T cells (CD3+HLA-DR+)/μL in non-ICU	43	67	39
Activated T cells (CD3+HLA-DR+)/μL in ICU	66	90	131
p	*		*
Activated suppressor T cells (CD3+CD8+HLA-DR+)/μL in non-ICU	23	30	20
Activated suppressor T cells (CD3+CD8+HLA-DR+)/μL in ICU	38	58	94
p			*
Regulatory T cells (CD3+CD4+CD25+CD127low+)/μL in non-ICU	33	48	37
Regulatory T cells (CD3+CD4+CD25+CD127low+)/μL in ICU	19	18	18
p	*	*	
Naïve regulatory T cells (CD45RA+CD3+CD4+CD25+CD127low+)/μL in non-ICU	7,6	11	11
Naïve regulatory T cells (CD45RA+CD3+CD4+CD25+CD127low+)/μL in ICU	3	2,7	2,9
p	*	*	*
Induced regulatory T cells (CD45RO+CD3+CD4+CD25+CD127low+)/μL in non-ICU	25	35	25
Induced regulatory T cells (CD45RO+CD3+CD4+CD25+CD127low+)/μL in ICU	16	15	14
p	*	*	
TCRα/β/μL in non-ICU	727	924	861
TCRα/β/μL in ICU	406	467	627
p	*	*	
TCRγ/δ/μL in non-ICU	42	43	57
TCRγ/δ/μL in ICU	9	13	16
p	*	*	
B cells (CD19+CD20+)/μL in non-ICU	387	490	297
B cells (CD19+CD20+)/μL in ICU	236	235	325
p	*	*	
NK cells (CD16+CD56+)/μL in non-ICU	171	194	87
NK cells (CD16+CD56+)/μL in ICU	52	50	85
p	*	*	

* statistical significance ($p<0.05$) between studied groups
