# Supplementary figures and images for: Immunosuppression as a Hallmark of Critical COVID-19: Prospective Study
Source: Cells. 2021 May 23;10(6):1293. doi: 10.3390/cells10061293 (PMC8224622; doi:10.3390/cells10061293)

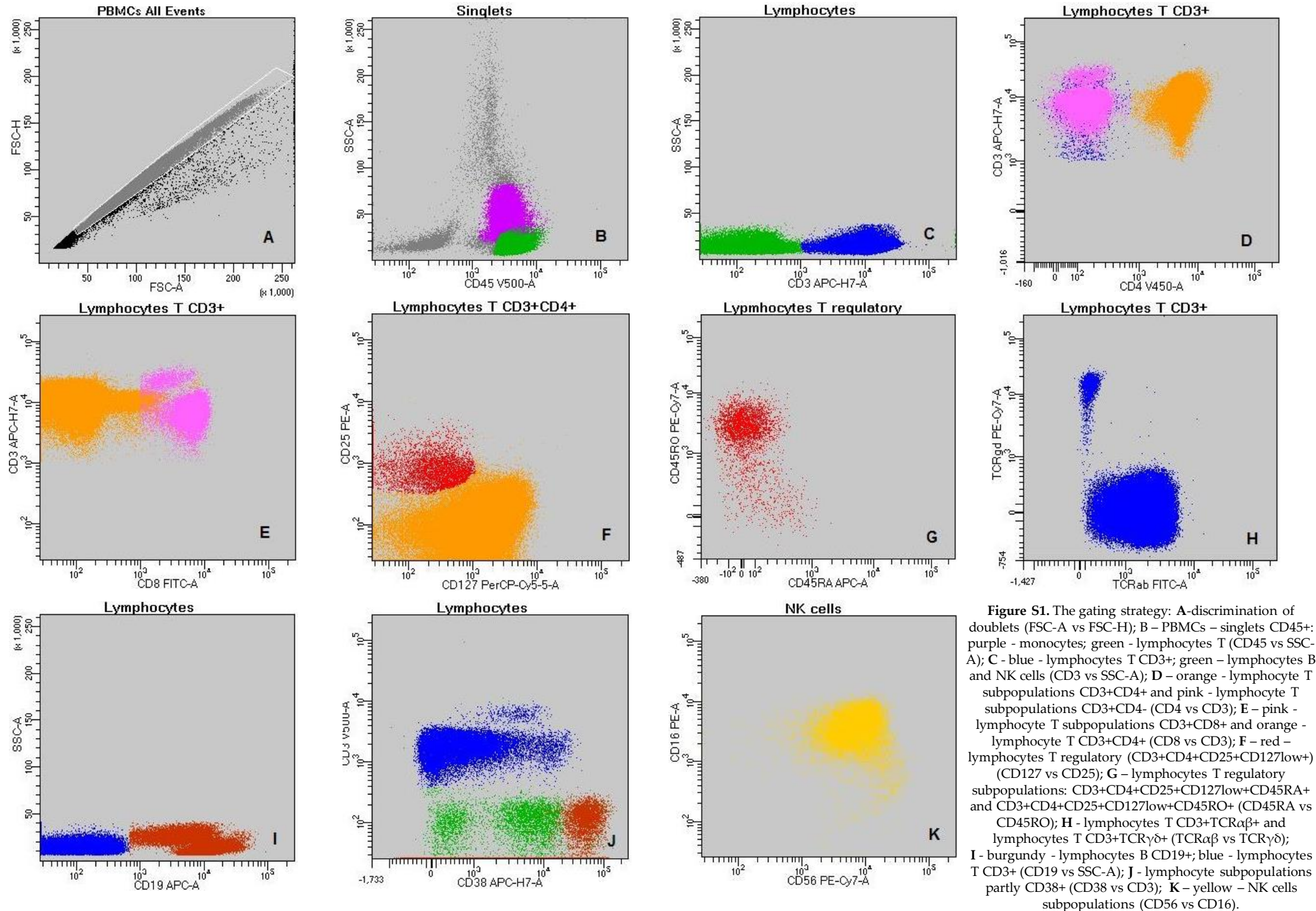

Supplement: Supplementary file 1 [file cells-10-01293-s001.zip › Supp fig 1.pdf]

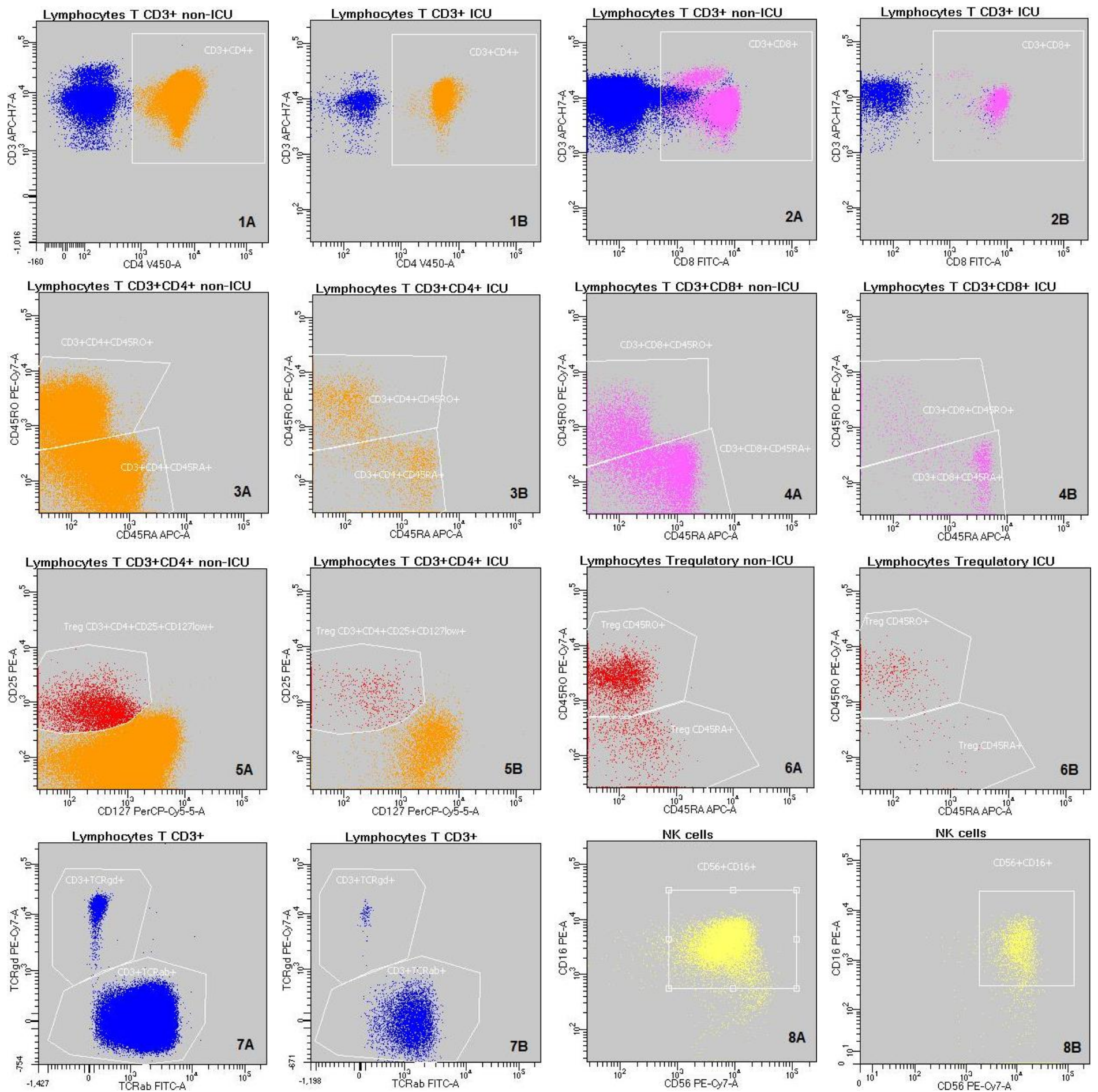

Supplement: Supplementary file 1 [file cells-10-01293-s001.zip › Supp fig 2.pdf]
